# Illusionism, Moore, and Chalmers

**DOI:** 10.3389/fpsyg.2024.1449314

**Published:** 2024-11-20

**Authors:** Evgeny V. Loginov

**Affiliations:** Faculty of Philosophy, Moscow State University, Moscow, Russia

**Keywords:** Moore, Chalmers, consciousness, illusionism, the Moorean argument, proof of an external world

## Abstract

In 1939, G. E. Moore presented his famous proof of an external world. In 2018, David Chalmers published his Moorean argument against illusionism. In 2022, Chalmers argued that Moore’s original argument was wrong. In this paper, I will try to defend the original Moore’s argument against Chalmers-style criticism, and show that Chalmers’s Moorean argument against illusionism cannot refute illusionism.

## 1 Introductory remarks

The *hard problem of consciousness* aims to explain why phenomenal consciousness exists (Chalmers, 1995, 1996, 2022). *Phenomenal consciousness* refers to the subjective, first-person experience characterized by *qualitative* features. It is often described using expressions like ‘what-it-is-likeness’. *Illusionism* is the view that phenomenal consciousness does not exist, but that we are under the illusion that it does (Dennett, 1991). However, many philosophers argue that illusionism is false (Searle, 1992; Kripke, 1980; Frances, 2008, p. 241; Nida-Rümelin, 2016; Strawson, 2017). The rejection of illusionism is known as *qualia realism*, which asserts the existence of phenomenal consciousness.^1^

*The Moorean argument* is a key objection to illusionism. It begins with (1) the evidence for the existence of phenomenal properties (such as pain) and (2) the illusionist denial of these properties. The argument concludes that (3) illusionism must be false. The name of the argument draws an analogy with G.E. Moore’s proof of an external world, and I will refer to it as *Moore’s argument*.

## 2 Arguments in favor of illusionism

Some arguments supporting illusionism are based on broad methodological considerations. Illusionists argue that a world without phenomenal properties is ontologically simpler, thus meeting *the criterion of simplicity*. If illusionism is true, it eliminates the hard problem of consciousness and potentially resolves the issue of mental causality. This can be referred to as the argument from theoretical *elegance*.

Another line of reasoning for illusionism is empirical. Illusionists point out that there is no single structure in the brain that could serve as the physical ‘location’ where all conscious data is unified. Furthermore, they highlight that no experiments within cognitive science explicitly address phenomenal data.

More specific arguments also support illusionism. For instance, Daniel Dennett argues that realism leads to an infinite regress of observers, or *homunculi*, observing each other: I perceive my qualia, I perceive how I perceive my qualia, I perceive how I perceive how I perceive my qualia, and so on, which is absurd (Dennett, 2013). Dennett also presents the problem of our inability to unambiguously identify qualia, which we supposedly know directly and infallibly. For example, I drank my usual morning coffee today, but this time I did not enjoy it. Dennett argues that there is no intrinsic fact that could tell me which of the following scenarios occurred: (1) the taste of the coffee has remained the same, but my preferences have changed, or (2) the taste is the same, but my sensory system has malfunctioned, misrepresenting the taste while I still like the original flavor. The lack of a decisive intrinsic fact leads to the inability to identify qualia. This argument can be called *the non-identification argument* (Dennett, 1988).

Frankish (2021) presents *an argument from the anomalousness of phenomenal consciousness.* The core of this argument lies in highlighting the radical difference between phenomenal consciousness and everything else in nature. It is clear that there are — or may be — many strange phenomena in the world: the sum of all natural numbers, the human need for sleep, chemical bonds of molecules (Frenking and Krapp, 2007), ball lightning, dark matter, the origin of viruses, Saturn’s rotational speed, the neurobiology of yawning, and more. While these phenomena are remarkable, and may even seem anomalous, phenomenal consciousness is fundamentally distinct from them.

The key distinction is that consciousness is inherently private, accessible only from a first-person perspective. In contrast, all other hypothetical or real anomalous phenomena of nature, despite their extraordinary nature, are either publicly observable or theoretical constructs based on publicly observable data. From the perspective of its proponents, phenomenal consciousness is neither of these.

Another argument in favor of illusionism was offered by his opponent David Chalmers (Chalmers, 2018). Since Frankish, François Kammerer, and other illusionists agree with this argument, it can also be used. This is *the debunking argument.*

1. There is a correct explanation of our beliefs about consciousness that is independent of consciousness.
2. If there is a correct explanation of our beliefs about consciousness that is independent of consciousness, those beliefs are not justified.
3. Our beliefs about consciousness are not justified.

It is important to note that, according to Chalmers, explaining beliefs is an “easy” problem, not a “hard” one. Therefore, it is theoretically possible to explain beliefs about consciousness without directly referencing consciousness itself.

A realist might respond to the debunking argument by claiming that they know consciousness directly, and thus their belief in its existence is more reliable than even the most reasonable criteria or theories, which are based on public data—that is, data received indirectly. The illusionist, however, counters that introspection, through which the realist claims to know their qualia, is a system of representation. Like any representational system, it is prone to error. Since introspection concerns qualia, entities that do not fit into the physical worldview, we must acknowledge that we are systematically mistaken about them. When it *seems* that there is something *what-it-is-like* to experience pain, in reality, there is no such thing (Chalmers, 2018; Stoljar, 2020b; Goff and Frankish, 2023).

In light of this, realists must provide a response to illusionists. They cannot merely assert that they know qualia exist, as illusionists can easily challenge this claim. Realists must not simply repeat their thesis but instead offer a supporting argument. This places them in a difficult position because what illusionists deny and realists affirm is often perceived as self-evident by many. Proving something self-evident, however, is notoriously challenging.

Realists typically adopt two strategies in response to illusionist criticism. First, they may attempt to defend the claim that they have *direct awareness* of their consciousness and that introspection is transparent, revealing the essence of conscious states. This *acquaintance* has such epistemic weight that it makes statements about consciousness more reliable than any counterarguments. This approach requires a robust theory of introspection. Second, they may provide a *argument* for realism. This article explores one possible way to pursue the latter approach in defending realism against illusionist arguments.

## 3 Analysis of the Moorean argument

The most well-known argument against illusionism is the so-called *Moorean argument*, coined by Chalmers. It consists of two premises and a conclusion:

1. People sometimes feel pain.
2. If illusionism is correct, then no one feels pain.
3. Therefore, illusionism is not true (Chalmers, 2018).

Chalmers argues that premise (1) “seems obviously true.” Premise (2) follows from the claim that the phrase “feel pain” refers to a conscious experience, combined with the fact that illusionism denies the existence of conscious experience (Chalmers, 2018, p. 53). Chalmers also notes that the terms “conscious experience” and “subjective experience” are synonymous with “phenomenal consciousness,” and that phenomenal properties are “what-it-is-like” properties (Chalmers, 2018, p. 6). For a similar argument, see (Nida-Rümelin, 2016).

The Moorean argument is not only supported by anti-physicalist philosophers like Chalmers but also by non-anti-physicalist scholars, including Stoljar (2021), Stoljar (2020a), Stoljar (2020b), Schwitzgebel (2014), and Schwitzgebel (2022). Stoljar, for example, presents a slightly different formulation of the Moorean argument:

1. I perceive a red object.
2. If I perceive a red object, then illusionism is false.
3. Therefore, illusionism is false.

An illusionist might argue that the first premise of the Moorean argument already assumes the falsity of illusionism, making it *a question-begging fallacy.* However, Stoljar contends that, in an important sense, no such fallacy occurs here. He compares this to the classic Moore’s argument: Moore can know that he has hands independently of any philosophical position, relying instead on perception or common sense. Thus, Moore does not presuppose the falsity of skepticism or idealism. According to Stoljar, the same applies to the Moorean argument about consciousness. He argues that one can know they are in pain or see a red object without requiring external evidence, based on the thesis that consciousness is transparent to introspection. This premise is not evidential; it does not depend on external support or theoretical justification (Stoljar, 2019; Stoljar, 2020a). Since one can know the first premise – that they see a red object or feel pain — without assuming that illusionism is false, *no question-begging fallacy is present.*

Kammerer refines the illusionist’s counterargument by further analyzing the comparison between Chalmers’s Moorean argument and what he considers Moore’s “standard” argument (Kammerer, 2022). He suggests that Moore’s standard arguments are effective against *philosophical* positions, but not *scientific* ones. For example, one can use a Moorean-style argument to refute skepticism, but not to challenge scientific theories like theory of relativity and prove the existence of absolute simultaneity. We cannot rely on common sense to critique modern cosmology by asserting, for instance, that the Sun is the size of the Moon (as it seems). Kammerer argues that illusionism is based on a blend of philosophical and scientific arguments, meaning that a standard Moorean-style argument is ineffective against it.

To justify the first premise of the Moorean argument, the realist must rely on a more rigorous type of reasoning, which Kammerer terms the “super-Moorean” approach. In this case, premise (1) is more robustly supported than in the standard Moore-style argument, making it potentially more persuasive than scientific arguments and, as some realists claim, even more compelling than the thesis about the existence of the external world, as noted by Goff et al. (2022). However, illusionists can employ a second-level debunking argument against the intuition supporting the super-Moorean certainty of the first premise. This contrasts with the first-level debunking argument, which concerns intuitions about the phenomenality of pain or seeing red.

In response, the realist could claim that they possess a super-Moorean certainty about the super-Moorean certainty of phenomenal pain, constructing a second-level super-Moorean argument in favor of this first-level certainty. The illusionist could then formulate a third-level debunking argument, and this exchange could, in theory, continue indefinitely. Kammerer’s key point is that at some point in this escalating process, “the friends of consciousness *will not be able to pull off one of their key moves anymore*” (Kammerer, 2022, p. 18). That is, at a certain level (n), it will no longer be obvious that the realist has the required unique epistemic relation at the previous level (n-1). At this point, the realist’s claim to super-certainty will cease to be part of common sense.

When faced with this, the realist may resort to invoking not only intuition but also a controversial philosophical theory, such as the epistemology of acquaintance, to defend their certainty at the first level. Kammerer suggests that each of these controversial theories, which are necessary to uphold the truth of the key premise in the Moorean argument, may entail the falsity of illusionism. Consequently, defending the key premise of the Moorean argument in this way ultimately begs the question. Therefore, Kammerer argues, the Moorean argument does not succeed.

Like Frankish, Kammerer draws a parallel between the acceptance of illusionism and other scientific revolutions. He compares illusionism to shifts in cosmological understanding during the Modern era or the rejection of absolute simultaneity after Einstein’s theory of relativity. In these cases, we did not abandon the notions of the Sun and Earth or of moments in time as existing – we simply altered our understanding of how they exist. Kammerer argues that we should approach consciousness in the same way: rather than denying its existence, we need to revise our theory of how it exists.

I am not convinced that there are scientific or philosophical arguments for illusionism that match the persuasiveness of the arguments against absolute simultaneity, the reasons humanity abandoned geocentrism, or the theory that stars are nails embedded in the sky. While there may be reasons to question the reality of qualia – such as the lack of evidence for a singular “location” of consciousness in the brain – this observation appears compatible with nearly every significant ontology of consciousness, from soul theory to eliminativism. I doubt that we should treat the arguments from simplicity, elegance, the homunculus fallacy, non-identification, anomalousness, and debunking as being scientific in the same sense as Einstein’s or Copernicus’s arguments. This is a subtle and somewhat confusing issue. One might argue that the arguments of Galileo or Einstein, like those of Dennett and Frankish, were speculative thought experiments. However, in addition to their speculative arguments, scientists like Galileo and Einstein made specific predictions that were later empirically confirmed. Their theories became embedded within broad research programs in astronomy and other natural sciences, significantly shaping the work of empirical scientists. Illusionism, by contrast, offers nothing of comparable impact.

Kammerer might respond to this line of reasoning in at least two ways. First, he could argue that there are examples in which two physical theories are empirically equivalent, yet we choose one over the other based on philosophical principles like simplicity and elegance – even if the rejected theory aligns more with common sense. I would agree with Kammerer (2022) and Rinard (2013) that it is, in principle, possible to prefer Einstein’s theory solely on grounds of simplicity or elegance. In that case, the argument against absolute simultaneity would not be purely scientific, but also philosophical – thus making the illusionists’ arguments comparable to Einstein’s. However, we might still argue that the illusionists’ case does not enjoy the same clear advantage as Einstein’s, not based on empirical shortcomings, but because, unlike the case of relativity theory, it is less clear whether simplicity and elegance unequivocally favor illusionism. Moreover, other reasons might carry more weight than these general criteria in this context.

Second, it could be argued that contemporary scientific theories of consciousness that do not reference qualia make predictions and that the simplest interpretation of these theories is illusionist. From this perspective, one might claim there are empirical data supporting illusionism. However, many empirical approaches to consciousness *do* address qualia (see: Lamme, 2003; Tononi, 2004; Prinz, 2012; Anokhin, 2021; Seth, 2021; Block, 2023).

If the illusionists’ arguments fail to reach the same level of reliability as Einstein’s, then the demand to replace Moorean certainty with super-Moorean certainty seems questionable. As such, the invocation of scientific revolutions as a critique of the Moorean argument appears to be dialectically weak. That being said, I acknowledge that philosophy can, in principle, overturn common sense judgments. However, in this particular case, I do not find the grounds for such a refutation to be sufficiently compelling.

Regardless of whether this reasoning concerning science and philosophy holds, I propose a different approach to analyzing the Moorean argument. Kammerer, like Chalmers, assumes that the thesis of phenomenal consciousness forms part of common sense (though Chalmers affirms it and Kammerer denies it). However, I argue that the existence of phenomenal consciousness is not a part of common sense.

Therefore, I suggest we re-examine the Moorean argument from a different angle. My approach, similar to that of Stoljar and Kammerer, involves comparing Moore’s original argument with the Moorean argument against illusionism. Assessing an argument, unless it is purely a question of logical validity, often requires comparing it to other arguments or some philosophical standard of justification. The key question is whether Moore’s original argument provides an appropriate benchmark. This remains a matter of debate (see, for instance: Moore, 1993; Klemke, 1969; Schaffer, 2009; Ichikawa, 2017; Schwitzgebel, 2024).

Some philosophers contend that Moore presupposes what he aims to prove. Chalmers, for example, critiques Moore’s original argument as question-begging and inadequate to address the problem of simulation (see Chapter 4 of Chalmers, 2022, pp. 79–80). However, I side with Stoljar in maintaining that Moore’s argument is not question-begging. It is valid, sound, and successful. Moore’s argument does not specify the content or nature of the external world. The world could, for instance, consist of simulated entities, which Chalmers acknowledges as “external” in his response to skepticism. Therefore, this defense is also available to Moore.

However, I will begin with a more modest claim: Moore’s argument is sufficiently effective in supporting belief in the existence of the external world. The task is to determine whether the Moorean argument against illusionism provides at least the same level of certainty regarding the existence of phenomenal consciousness as Moore’s argument provides for the external world. My approach differs from Kammerer’s in that I will not draw any conclusions about scientific revolutions.

Before proceeding further, I must clarify an important point. Chalmers may argue that the obviousness of the Moorean argument far exceeds that of Moore’s original argument, making it unnecessary to show that the former is at least as effective as the latter. Indeed, it might seem that we cannot doubt the existence of phenomenal consciousness in the same way we might doubt the existence of the external world. However, the only way I see for Chalmers to justify this privileged epistemic position is by appealing to a special capacity of acquaintance as a source of infallible knowledge^2^ of mental life. It should be acknowledged that if a realist invokes such a special capacity, this significantly shifts the debate. Nonetheless, Chalmers’ Moorean argument is notable for attempting to refute illusionism without relying on the acquaintance thesis (although Chalmers seems to admit to its relevance). In this discussion, I will address the realism versus illusionism debate without invoking the question of whether or not we possess a special, infallible ability to access qualia.

## 4 Moore’s argument and the Moorean argument

### 4.1 Choice of wording

There are various ways of presenting the Moorean argument, and while Stoljar’s and Chalmers’s versions are essentially equivalent, any assessment of one should apply to the other. For convenience, I will use Stoljar’s formulation, as it allows for easier comparison with the version of Moore’s argument that I will employ. However, to maintain consistency with the tradition in the philosophy of mind (Shoemaker, 1996, p. 226), I will replace Stoljar’s example of red perception with an example involving pain.

Determining the precise version of Moore’s argument, however, is more challenging. If we take the text of “Proof of an External World” [first publication 1939; Moore, 1993] literally, Moore presents the argument as follows:

1. Here is one hand.
2. And here is another.
3. Therefore, at this moment, two human hands exist.

Moore himself considers (3) to be the conclusion of his argument, aiming to demonstrate that it satisfies the three criteria for a sound argument: (i) the premises differ from the conclusion, (ii) the premises are known to be true rather than simply believed, and (iii) the conclusion follows logically from the premises. However, in this form, it may be unclear how this proves the existence of an external world.

Moore seems to think the work is complete because he has previously argued that statements like “At this moment, there are two human hands” entail the claim that “There are at least two external objects.” His reasoning appears to be the following: Suppose I perceive an object in space, such as a soap bubble. *If I assert “This is a soap bubble,” I imply that it is logically consistent to claim that the perceived object existed before my perception and will continue to exist after I stop perceiving it*. Why? Because this is part of what it means to assert that the soap bubble is real rather than a hallucination.

Given this, we can reformulate Moore’s argument as follows:

1. There are two hands.
2. If there are two hands, then external objects exist.
3. Therefore, external objects exist.

This formulation, I believe, also satisfies Moore’s three requirements for a sound argument.

Before comparing Moore’s argument to the Moorean argument, it is essential to clarify against whom Moore’s argument is directed (just as the Moorean argument is directed against illusionism). I will focus on how I interpret the aim of Moore’s argument, as the historical Moore’s purpose remains somewhat ambiguous.^3^ From my perspective, Moore’s argument is effectively employed against the following thesis:

*Moore’s argument’s aim:* There is no external world (or external objects).

This thesis could arise from two distinct philosophical stances. First, it might stem from *skepticism*. A skeptic might argue that we lack sufficient justification for believing in the existence of an external world and that we should not hold beliefs for which we have no compelling reasons. Therefore, the skeptic refrains from believing in an external world. So, she rather thinks that there is no external world.

Second, this thesis could be a consequence of *solipsistic idealism*. A solipsist holds that there is no external world because everything exists solely in her mind. However, there is another form of idealism that posits reality as fundamentally spiritual and not reducible to individual perception. In this form of idealism, external objects that I do not perceive can (at least in a doxastic sense) exist in the perceptions of others, including, perhaps, in the perception of God. Such idealism is compatible with Moore’s conclusion that an external world exists. Historically, Moore addresses this broader form of idealism in a different argument.^4^ However, the idealism under consideration in this paper is restricted to solipsistic idealism.

### 4.2 The first premise

The thesis that there are two hands, or that I am in pain when shown two hands or experiencing pain, represents what I believe Richard Cartwright aptly describes as “irresistible objects of belief” – if anything deserves such a description. To assert this does not imply that we know how to prove we know the truth of the first premises. Perhaps this is a complex epistemological issue, or perhaps we simply know that Moore has hands because he is showing them to us, and there is no reason to distrust our perception. What matters is that we know Moore has hands when they are presented to us, not how we know it. The same is true of pain: we know we have hands (if we do) and that we are in pain (when we are), simply from experience, not from an inference drawn from some philosophical theory.

The skeptic may, of course, tell Moore that they will not accept the existence of hands. However, this is a dialectically meaningless stance when Moore is *showing* the skeptic his hands.^5^ The skeptic can be invited to address any reasonable doubts they may have. They can touch the hands of Moore, examine them carefully, consult with a psychologist to assess their memory and cognitive abilities, and so on. Of course, the skeptic might still insist that their epistemic standards are such that they cannot concede that Moore knows he has hands. Moore himself responded to this by saying, “You might as well suggest that I do not know that I am now standing up and talking – that perhaps after all I’m not, and that it’s not quite certain that I am!” (Moore, 1993, pp. 166–167).

Perhaps skeptics or idealists might claim that the individual standing before them, declaring they are standing, does not know what they are saying or even that they are standing. I have personally encountered bold philosophers who are willing to defend such theses, though they are not always driven by traditional skeptical or idealist concerns. In my experience, they are often inspired by the Parisian philosophy of the latter half of the 20th century. In such cases, the best course of action for a proponent of Moorean arguments may be to conclude the serious discussion and transform it into a lighthearted, local joke, gently pointing out to the skeptic the extreme improbability of their claims at every opportunity. The denial of the first premise of Moore’s argument is only possible when one adheres to unrealistic standards of justification, which there is no compelling reason to adopt. Therefore, I suggest that a gentle joke is the most appropriate response for an open-minded philosopher in such situations.

Does the first premise of the Moorean argument meet the standard set by the first premise of Moore’s original argument? Chalmers believes that his premise is obviously true, and illusionists may agree. However, they might ask for clarification: in what sense are we using the word “pain” here? If we mean *phenomenal* pain, then, of course, there is nothing obvious about it for the illusionist. The argument becomes question-begging since illusionists directly deny the existence of phenomenal properties. Chalmers responds by asserting that when we typically speak of pain, we refer to some painful experience, and when someone experiences pain, “almost by definition,” there is something it is like to feel that pain (Chalmers, 2018, p. 53). Illusionists, however, cannot accept this “almost by definition” claim.

Nevertheless, illusionists must acknowledge that we might think there is something it is like to feel pain. At the very least, I can have the illusion that I am in pain (in the phenomenal sense). I believe that the illusionist will lose dialectical persuasiveness if they prohibit us from accepting the first premise based on their theory. The realist demands that the first premise be understood in the sense that “I’m in pain” *actually* refers to a phenomenal property. The illusionist might agree with this, provided the realist is willing to remove the word “actually” from the statement. It seems that the illusionist’s position does not prevent them from agreeing that people often feel pain, that pain can have certain properties, and that the term “pain” seems to refer to some property that a person observes “from the inside.” Pain may seem to be something inherent, non-physical, and perhaps ineffable. The illusionist can agree with this interpretation but cannot concede that such a property truly exists. They cannot admit that the term “pain” actually refers to the qualia of pain. Simply put, the illusionist may agree that we understand that we feel pain without presupposing realism about qualia. After all, I can know I am in pain through introspection, not through inference from any philosophical theory.

The situation here is dialectically similar to that of Moore’s argument. A skeptic or idealist might accept Moore’s first premise, intending to provide their interpretation. Yet, it is dialectically unprofitable for any opponent of Moore’s argument to outright deny that Moore has hands. The illusionist finds themselves in a comparable position: they can accept the first premise, hoping to later interpret it in line with illusionism.

In short, the first premise of the Moorean argument is quite reliable.

### 4.3 Second premise

The second premise of Moore’s argument relies on the *Independence Thesis*, which can be formulated as follows:

**The Independence Thesis**: objects like hands can exist even if no one perceives them.

Moore also suggests that we can accept the following *definition of external objects*:

**The Definition of External Objects**: external objects are those whose existence is logically independent of perception.

Skeptics and idealists may accept this Definition but reject the Independence Thesis. However, it seems unlikely that much can be convincingly derived from this rejection. The strength of Moore’s argument lies *in the fact that the Independence Thesis is part of common sense.*

Of course, common sense is not infallible. In practice, it is a complex process, and we must acknowledge that common sense can be mistaken or biased and that there are contexts where it is not directly applicable. However, this does not mean we have sufficient reason to abandon it completely in favor of some allegedly better model of thinking. To conclude that an alternative model is more advanced, we would need criteria derived from generalizing our past experiences, of which common sense is the quintessence. Thus, to recognize a common-sense belief as false, we require strong reasons. Until such reasons are provided, common sense remains our starting point, as there is no alternative foundation for our inquiries.

Therefore, a reasonable skeptic or idealist should acknowledge the truth of the Independence Thesis and provide an interpretation within their system. The best-known way to reconcile the Independence Thesis with an idealist framework is to claim that things unperceived by humans exist in the perception of God. The skeptic, however, lacks such an easy solution. She might argue that while we accept the Independence Thesis in ordinary life, we can question it within the domain of philosophy. The skeptic asserts this because the realists cannot explain *how* they know the Independence Thesis is true. Yet, the skeptic must provide reasons for believing we do not know the truth of the Independence Thesis. So far, I believe no skeptic has provided sufficient reasons. Therefore, the realist has the right to employ the Independence Thesis, and Moore’s argument remains sound.

Now, does the second premise of the Moorean argument achieve a similar level of certainty? We shall see. I propose that a realist should accept something akin to *the Qualitative Thesis*:

**The Qualitative Thesis**: if there is pain, there is a what-it-is-likeness of pain.

As we have seen, Chalmers believes he can justify this thesis (or a similar one) “almost by definition.” While anyone is free to propose definitions, others are not obliged to accept those that seem substantive rather than merely formal. The illusionist might argue that, for her, “feeling pain” simply means “realizing a particular functional pattern” or something along those lines, where nothing like “what-it-is-likeness” is involved. Hence, the realist cannot obtain the Qualitative Thesis so easily.

The realist needs to demonstrate that the Qualitative Thesis is at least as credible as the Independence Thesis. I am not convinced that this can be accomplished. The Qualitative Thesis is not a conceptual truth. The realist might contend that “if there is pain, there is something it is like to be in pain.” While this is indeed seeming as a conceptual truth, it can be interpreted either realistically or illusionistically. An illusionist might say that they can experience varying intensities of pain – one sharp and another more akin to an itch, for instance.

Chalmers cannot claim that the Qualitative Thesis is a conceptual truth. The denial of a conceptual truth would be self-contradictory, but Chalmers himself acknowledges that illusionism is a coherent doctrine (Chalmers, 2018, p. 52). His argument against illusionism is not that it is self-contradictory, but that it is false, as demonstrated by the Moorean argument. Chalmers also finds illusionism highly counterintuitive, but this point is irrelevant to the current discussion.

*The best option for Chalmers, in my view, is to argue that the Qualitative Thesis is a part of common sense.* This would place him in a similar position as the proponent of Moore’s argument. However, I am not convinced this can be established.

I will provide three reasons for skepticism, but first, I must offer one clarification. The realist might demand a reformulation of the Qualitative Thesis to refer not to “what-it-is-likeness” but to “feelings,” “subjectivity,” or “experiences”.^6^ But why should the illusionist not agree that when someone feels pain, they are experiencing pain? As long as the realist does not insist that these feelings or experiences cannot be described in functional terms, the illusionist can accept the occurrence of such feelings, experiences, and subjective states.

The realist might object here, claiming they do not understand the illusionist’s position: if the illusionist accepts the existence of experiences, why does she not accept the existence of consciousness in the realist sense? The answer is that the illusionist denies the existence of *phenomenal* consciousness but not consciousness in general. If the realist fails to grasp this distinction, then they may not fully understand their claims either.

### 4.4 The qualitative thesis and common sense

#### 4.4.1 “What-it-is-likeness” as a philosophical term

The Qualitative Thesis, unlike the Independence Thesis, appears to incorporate what might be considered a philosophical term. One reason the Independence Thesis can be associated with common sense is the absence of specialized philosophical terminology in its formulation. Chalmers suggests that his argument involves the “ordinary sense” of the word *feel*, as in “feel pain” (Chalmers, 2018, p. 53). However, I am not entirely convinced. It seems to me this could be an empirical question, one that might be resolved through, for instance, corpus analysis specific to each language. It seems plausible that in natural languages, word usage is mixed: part of the sample will likely refer to phenomenal pain, another to functional pain, and a third to normative pain [these types of pain are categorized in (Kammerer, 2022)]. Speculating further, I would propose that pain is most often discussed in a normative context (pain is undesirable, no one should inflict pain without justification, etc.). Nonetheless, this is an empirical matter, one for which philosophers could collaborate with linguists to achieve a more precise answer (see, for example, Wierzbicka, 2019; for further discussion, see Chalmers, 2020). Care should be taken here: maybe the mere tendency (or lack thereof) for people to attribute phenomenality to pain does not necessarily inform us about pain itself, any more than people’s tendency to attribute functionality to speed tells us something definitive about speed itself.

In the sense that “what-it-is-likeness” is used in the philosophy of mind, it is evidently a term, as indicated by the suffix “-ness,” the hyphenated spelling, and the use of quotation marks. Of course, these markers are neither necessary nor sufficient for terminologization; rather, they serve as indicators of the unique status of these words and their potential terminological role.

In this respect, nothing discussed in the Independence Thesis qualifies as a technical term. While “perception” may indeed be classified as a philosophical term, the thesis itself can easily be reformulated without it. For instance, my hands (and similar objects) will continue to exist even if I am unable to see, hear, feel, smell, or taste them. I doubt, however, that Chalmers could achieve a comparable reformulation for the Qualitative Thesis.

*There appears to be a reason for this. Chalmers requires a term – one that we might substitute for “what-it-is-likeness” – to construct his zombie argument.* If this is the case, then the meaning of such a term diverges considerably from common sense, given that philosophical zombies themselves are far from intuitive. This point holds even if we assume zombies to be conceivable (a thesis I am inclined to accept).

I must acknowledge that it is possible to teach someone what realism signifies with respect to qualia and to convey the qualitative aspect of experience. This is something I regularly do in teaching philosophy of mind. Through such instruction, I believe we cultivate new concepts within the students, enabling them to articulate beliefs that are absent in pre-theoretical common sense. Nevertheless, some might argue that teaching the zombie argument and differentiating between psychological and phenomenal concepts of consciousness merely helps an individual articulate beliefs they already hold, but cannot express in ordinary language or illustrate with common examples.

In my view, this argument is flawed. I find it difficult to comprehend what it means to possess awareness of phenomenal consciousness without the ability to express it. Of course, a child may experience joy, for example, without yet knowing what it is called and without the ability to offer examples, due to age. However, joy appears far more commonplace than any concept through which one might construct a zombie argument. Perhaps my imagination is simply insufficiently abstract.

#### 4.4.2 The qualitative thesis and understanding

It is unlikely that any reasonable person would encounter difficulty grasping the meaning of the Independence Thesis. We assume, for a certain class of objects, that they can exist independently of our perception. For instance, when I place a kettle on the stove, leave the kitchen, and go to my room to write this article, I presume that the kettle – although unobserved by any human person or my cat – continues to exist on the stove and, in due time, the water within it will boil.

Certainly, this common-sense judgment is compatible with the statement that God might perceive my kettle even if no human person or pet can see it (as Berkeley seemingly believed). However, this assumption appears superfluous. One might also suggest that my common-sense belief should be interpreted in a hypothetical context: upon returning to the kitchen, I would find the kettle there. Such an interpretation, however, does not entail that I believe the kettle exists as long as it remains unseen. This seems not to be the case. I firmly believe it is on the stove, even though no one can see it, and this strikes me as a matter of common sense rather than hypothetical metaphysics.

*Concerning the Qualitative Thesis, however, it is far more challenging to provide such an everyday example.* This, in itself, is not a problem; we can understand ideas that are not directly related to our daily experience. *We can indeed comprehend* the arguments of philosophers who are known for their complexity, such as Plotinus, Duns Scotus, Schelling, Derrida, Dummett, and Stalnaker, *even if* their ideas do not belong to the realm of common-sense truths. We *should not expect* these esoteric ideas to align with the truths of common sense. Admittedly, the Qualitative Thesis is not among the most abstruse formulations in philosophy. It is far removed from the pinnacles of obscurity in this regard. However, to grasp the type of thought it intends to convey, one typically requires some familiarity with the philosophy of mind. In my experience, even philosophy students who are introduced to contemporary philosophical discourse do not immediately learn how to apply the term “what-it-is-likeness” accurately and contextually. Such difficulties do not arise with the independence thesis.

Certainly, any further exploration and clarification of the ideas presented in this paragraph would greatly benefit from the work of a philosopher who is willing to engage in experimental thinking.

#### 4.4.3 The Realist’s dilemma

Ryle (1953) introduces a useful distinction between “the use of ordinary language” and “ordinary linguistic usage.” According to Ryle, “use” refers to a technique, knack, or method – learning it involves learning how to perform a task, rather than discovering sociological generalities. In contrast, “usage” denotes custom, practice, fashion, or vogue, and the methods for uncovering linguistic usage are those of philology. In the first argument, I discussed *usage* more broadly; now, however, we may turn our attention to *use*.

The notion of “what-it-is-likeness” seems applicable in everyday contexts. Consider the following scenario: I visit a doctor, who asks about my symptoms. I respond that I am in pain.

Doctor: And what does it feel like?

Me: It feels like something is pricking me from the inside out.

This example is intended to illustrate that we can respond to questions such as “What is it like to feel it?” by comparing one experience to another. The realist faces a potential issue with this practice, however: all such responses seem reducible to functional, structural, or behavioral terms. What is conveyed in these answers, at least at first glance, lacks any qualities that are entirely private, inexpressible, immediate, self-evident, or intrinsically internal. Nevertheless, the realist is certainly justified in insisting that no verbal *expression* of pain can *fully* capture the experience itself.^7^ This could be termed *the inexhaustibility* of pain rather than its *ineffability*. The aspect of a specific pain experience that seems to persist – if we exclude everything that can be described – is what may serve as a plausible candidate for the “quale” of this pain.

*The realist’s challenge here is that any experience that can be expressed is subject to some form of illusionistic translation.* Furthermore, what is unspeakable is exceedingly difficult to convey in propositional terms. As a result, realists often resort to using demonstrative pronouns and italics to articulate aspects of pain, bringing their view closer to the Type-B physicalists’ approach – an alignment between the phenomenal and the indexical, which Chalmers notably opposes (Chalmers, 2018, pp. 21–22).

This situation places the realist in a dilemma. Either they describe their experience ordinarily, or they restrict themselves to a specialized practice, relying on demonstrative pronouns and emphatic markers like intonation or italics. Should the realist opt for the first approach, their descriptions seem susceptible to functional analysis. If, however, they choose the latter, they may avoid functional analysis and preserve the ontological uniqueness of qualia – but only at the cost of forfeiting claims that their mode of expression is ordinary. The term “what-it-is-likeness” thus assumes a quasi-philosophical status, indicating that the realist cannot expect the Qualitative Thesis to carry the same degree or type of credence and reliability as the Independence Thesis.

#### 4.4.4 An argument against Chalmers’ Moorean argument

1*. If the Moorean argument is persuasive, then premises (1) and (2) are matters of common sense.

2*. If no relevant, commonplace examples can be formulated that cannot be interpreted in an illusionistic framework, then premise (2) is not a matter of common sense.

3*. No relevant, commonplace examples can be formulated that cannot be interpreted in an illusionistic framework.

4*. Therefore, premise (2) is not a matter of common sense.

5*. Consequently, the Moorean argument is not persuasive.

This argument is valid. Premise 1* of my argument assumes that the premises of the Moorean argument could be empirical truths, conceptual truths, or truths grounded in common sense. Since this paper excludes defenses of realism about phenomenal consciousness that rely on theories of introspection (such as a theory of acquaintance), premise (2) of the Moorean argument cannot be considered here as an empirical truth. Realism about phenomenal consciousness may indeed be true due to certain theories of introspection, but this possibility falls outside the scope of the present discussion.

Moreover, premise (2) would not qualify as a conceptual truth, nor does it meet the criteria for a common-sense truth, as I argue in this paper. My three reasons for contending that the Qualitative Thesis does not align with common sense are as follows:

- If a claim cannot be unambiguously expressed in ordinary language, it is highly doubtful that it is part of common sense (see § 4.4.1).
- If it is exceptionally challenging to formulate an ordinary, everyday example of the thesis, it is highly doubtful that it is part of common sense (see § 4.4.2).
- The Realist’s Dilemma (see § 4.4.3).

Hence, Chalmers’ argument does not establish the falsity of illusionism.

## 5 Conclusion

I hope that my argument, showing that the Qualitative Thesis does not align with common-sense truths, demonstrate that a realist cannot justifiably assume the soundness of *Moore’s argument* extends to the soundness of *the Moorean argument*. This does not imply that the Moorean argument is flawed, nor that it cannot be formulated differently. It also does not preclude the possibility that the Moorean argument could be developed within an epistemological framework distinct from Moore’s. Furthermore, it does not suggest that there are no other ways to critique illusionism. I am hopeful that there are. My reasoning, if correct, indicates only that the Moorean argument does not achieve the level and type of persuasiveness that Moore’s argument possesses – and that Moore’s argument is, indeed, persuasive.

## Data Availability

The original contributions presented in the study are included in the article/supplementary material, further inquiries can be directed to the corresponding author/s.

## Footnotes

^1^Technically, one could distinguish between qualia and the phenomenal properties. For example, it might be argued that qualia are fundamentally non-representational and lack intentionality, while the phenomenal properties could be representational. In this view, one could reject qualia while denying illusionism and still affirm the existence of phenomenal consciousness. However, I follow Chalmers’s terminology, in which qualia and phenomenal properties are treated as identical (Chalmers, 1996). See also (Shoemaker, 1996, p. 121).

^2^An reviewer requested clarification regarding my use of the term “knowledge.” I do not adopt a specific, formal stance on the nature of knowledge. Generally, I accept the view that knowledge is justified true belief, with additional elements that address the challenges posed by the Gettier problem (Chalmers, 2012, p. 55).

^3^He wrote: “I did, then, just now, give a proof that there were *then* external objects” (Moore, 1993, p. 167). He also stated that he knew the premises of the argument. So, it seemed clear that he claimed to know that there are external things. In this reading, the aim of the argument is skepticism. But later he claimed that he had proved that it’s false that “There are no material things,” not that “Nobody knows for certain that there are any material things” is false (Moore, 1942, pp. 668–675). However, the distinction he is trying to make seems rather vague to me, as these statements seem to be related in an important sense. I will not delve into the historical hermeneutics of Moore’s text here.

^4^(Moore, 1903). Chalmers and Daniel Stoljar refer to this argument as the “relationality problem” for idealism. Chalmers attempts to demonstrate that this argument does not pose a serious challenge to cosmic idealism (Chalmers, 2017).

^5^We must distinguish between two types of skeptical objections to the first premise: a skeptic may argue that Moore does not know the first premise or argue that the first premise is circular. In this paper, I will focus on the former type of objection, assuming that enough has been said about the latter type (see Section 3).

^6^This idea was suggested to me by Chalmers.

^7^However, an illusionist could reasonably argue that such inexhaustibility is universal: one can endlessly find new ways to describe a sunset, for example, but this does not make it something ineffable.
